# TurboID-mediated proximity labeling technologies to identify virus co-receptors

**DOI:** 10.3389/fcimb.2024.1371837

**Published:** 2024-06-27

**Authors:** Bo Wang, Fan Yang, Wuqian Wang, Fei Zhao, Xiaofang Sun

**Affiliations:** ^1^ Guangdong Provincial Key Laboratory of Major Obstetric Diseases; Guangdong Provincial Clinical Research Center for Obstetrics and Gynecology; Guangdong-Hong Kong-Macao Greater Bay Area Higher Education Joint Laboratory of Maternal-Fetal Medicine, The Third Affiliated Hospital of Guangzhou Medical University, Guangzhou, China; ^2^ Research Center for Lin He Academician New Medicine, Institutes for Shanghai Pudong Decoding Life, Shanghai, China; ^3^ Bio-X Institutes, Key Laboratory for the Genetics of Developmental and Neuropsychiatric Disorders (Ministry of Education), Shanghai Jiao Tong University, Shanghai, China; ^4^ Jiaxing Maternity and Children Health Care Hospital, Affiliated Women and Children Hospital Jiaxing University, Jiaxing, Zhejiang, China

**Keywords:** proximity labeling, TurboID, co-receptors, Axl, SARS-CoV-2

## Abstract

Virus receptors determine the tissue tropism of viruses and have a certain relationship with the clinical outcomes caused by viral infection, which is of great importance for the identification of virus receptors to understand the infection mechanism of viruses and to develop entry inhibitor. Proximity labeling (PL) is a new technique for studying protein-protein interactions, but it has not yet been applied to the identification of virus receptors or co-receptors. Here, we attempt to identify co-receptor of SARS-CoV-2 by employing TurboID-catalyzed PL. The membrane protein angiotensin-converting enzyme 2 (ACE2) was employed as a bait and conjugated to TurboID, and a A549 cell line with stable expression of ACE2-TurboID was constructed. SARS-CoV-2 pseudovirus were incubated with ACE2-TurboID stably expressed cell lines in the presence of biotin and ATP, which could initiate the catalytic activity of TurboID and tag adjacent endogenous proteins with biotin. Subsequently, the biotinylated proteins were harvested and identified by mass spectrometry. We identified a membrane protein, AXL, that has been functionally shown to mediate SARS-CoV-2 entry into host cells. Our data suggest that PL could be used to identify co-receptors for virus entry.

## 1 Introduction

Historically, emerging and re-emerging viruses appear constantly, killing tens of millions of human lives (Lu et al., 2021). Currently, both climate change and intense globalization have created more favorable conditions for the spread of the viruses. It is probable that future outbreaks of new emerging viruses will become more frequent. Viruses are strictly intracellular parasitic organisms, and their life cycle is entirely dependent on the hijacking of cellular functions to facilitate their replication, which starts with the interaction between viral particles and receptors on the host cell surface, in particular the binding of the viral surface protein to the receptors expressed in host cells (Sieczkarski and Whittaker, 2005; Maginnis, 2018). Receptors serve as the primary mediators of viral tropism, the host range and transmissibility, and in many cases, receptor interactions occur in a seemingly programmed series of events utilizing multiple receptors (Sieczkarski and Whittaker, 2005). The study of the dynamic mechanism of the virus entry process is not only a fundamental scientific issue in viral infection, but also has important implications for vaccine design and entry inhibitor research.

Due to the fact that protein-protein interactions (PPIs) are mainly mediated by hydrogen bonds, salt bonds and hydrophobic forces, all of which have very short interaction distances, it is generally believed that interacting proteins must be close to each other. Proximity labeling (PL) is an emerging approach to study the spatial characteristics of proteins and PPIs in living cells, with unique advantages in the identification of proteins interacting with hydrophobic and low-abundance proteins, and the analysis of weak or transient PPIs, and on the temporal-spatial resolution (Yang et al., 2021). PL uses engineered enzymes, such as peroxidases (engineered ascorbate peroxidase 2 (APEX2) (Lam et al., 2015), horseradish peroxidase (HRP) (Kotani et al., 2008)) or biotin ligases (BioID (Choi-Rhee et al., 2004; Roux et al., 2012), BioID2 (Kim et al., 2016), BASU (Ramanathan et al., 2018), TurboID (Branon et al., 2018), miniTurbo (Branon et al., 2018)), that are genetically tagged to a protein of interest (Qin et al., 2021). TurboID was developed through error-prone PCR mutagenesis and directed evolution in yeast and exhibits faster labeling kinetics, allowing labeling durations as short as 10 min (Branon et al., 2018). While the interaction between virus particles and receptors is relatively weak, the action time is short, and the dynamic process is fast, thus TurboID-catalyzed PL has the potential to identify virus receptors or co-receptors.

Classical biochemical and immunological approaches use the high-affinity interactions between the viral surface and its cellular target in order to identify the virus receptor(s), such as virus overlay protein blot assay (Tayyari et al., 2011), co-immunoprecipitation assay (Li et al., 2003; Raj et al., 2013; Wang et al., 2021). PL has been widely used to study membrane-protein interactions (Arora et al., 2020; Szczesniak et al., 2021), but it has not yet been applied to the study of virus receptors/co-receptors. Here, we apply TurboID-catalyzed PL to identify the co-receptor in SARS-CoV-2 entry. TurboID-conjugated ACE2 was stably overexpressed in the SARS-CoV-2 poorly susceptible A549 cell lines, and then these cells were bonded with SARS-CoV-2 pseudovirus in the presence of biotin and ATP, which could initiate the catalytic activity of TurboID and tag adjacent endogenous proteins with biotin. Subsequently, the biotinylated proteins were harvested using streptavidin-coated beads, and identified by mass spectrometry. Mass spectrometry analysis revealed that a membrane protein, AXL, which has been reported to be a receptor for SARS-CoV-2, was significantly enriched in SARS-CoV-2 pseudovirus-binding cells. Our data suggest that PL could be used to identify virus co-receptors.

## 2 Methods

### 2.1 Cell lines and plasmid construction

HEK-293T (a human embryonic kidney 293T cell line, CRL-11268) and A549 (a human non-small-cell lung carcinoma cell line, CCL-185) were purchased from ATCC and cultured in Dulbecco’s modified Eagle Medium (DMEM, HyClone). The medium was supplemented with 10% fetal bovine serum (FBS, Gibco, Australia), 2 mM L-glutamine, 100 U/ml penicillin and 100 μg/ml streptomycin (Life Technologies, NY), and maintained at 37°C in a humidified atmosphere containing 5% CO_2_.

For plasmid construction, genes were amplified using Transtart FastPfu DNA polymerase (TransGen Biotech). The vectors and PCR product were digested by restriction enzyme and then separated on an agarose gel, purified using MiniBEST Agarose Gel DNA Extraction Kit (TaKaRa). Vectors and PCR product were then ligated using T4 DNA ligase.

### 2.2 Production of overexpression lentivirus and SARS-CoV-2 spike pseudovirus

In order to overexpress specific genes (TurboID-ACE2 or ACE2-TurboID or ACE2 or AXL) in A549 cells, transduction by lentivirus was adopted. For preparation of lentivirus overexpressing specific genes, HEK293T cells in T25 flasks were transfected with packaging plasmid psPAX2 (Addgene, 2250ng) and envelope plasmid pMD2.G (Addgene, 750ng) and specific transfer plasmids (3000 ng). After 60 h incubation, the supernatant was collected and centrifuged at 5,000 × g at 4°C for 10 min to remove cell debris, and then passed through a 0.45 μm filters with low protein binding membrane (Millipore), aliquoted and stored at -80°C. For overexpression of specific genes, lentivirus transductions were performed in the presence of 10 μg/ml polybrene.

SARS-CoV-2 spike pseudoviruses bearing mCherry-SARS-CoV-2 N were produced as previously described (Ou et al., 2020; Mi et al., 2023). Briefly, HEK293T cells were co-transfected with psPAX2, pLenti-mCherry-N, and the plasmids encoding spike (S) proteins of SARS-CoV-2 by using polyetherimide (PEI) (Beyotime). The supernatants were harvested at 60 h post transfection, centrifuged at 5000 × g for 10 min, and passed through 0.45 μm filter to remove cell debris. The titer of SARS-CoV-2 pseudovirus in the supernatants was determined using the Lenti-X qRT-PCR Titration Kit (Clontech) and converted to infectious units (IFUs) according to the manufacturer’s instructions.

### 2.3 Immunofluorescence

A549 cells were transduced with the lentivirus expressing specific genes, and the cells were split at 72 h post-transduction and seed on glass bottom dishes for another 24 h and then fixed with 4% paraformaldehyde (PFA) at room temperature for 15 min followed by permeabilization by 0.5% Triton X-100 for 10 min at 4°C. After blockade with 1% normal goat serum (Boster) in PBS for 1 h, the cells were incubated with indicated primary antibody for overnight at 4°C. Subsequently, the cells were washed three times with PBS and incubated with suitable secondary antibody and finally stained with DAPI. The dishes were observed using a Perkin Elmer UltraViewVox confocal microscopy under a 60× oil objective. While the A549 cells stably expressing ACE2-TurboID were first plated on glass bottom dishes, then the cells were infected with SARS-CoV-2 pseudovirus. At 48 h post infection, the cells were fixed with 4% PFA and stained with DAPI, followed by observation using confocal microscopy.

### 2.4 PL and liquid chromatography-mass spectrometry analysis

For each sample, A549 cells stably expressing ACE2-TurboID were grown as a monolayer in 100 mm dish. The cells at ~80% confluency were infected with the SARS-CoV-2 spike pseudoviruses at a multiplicity of infection (MOI) of 2 or left uninfected for 15 min and at the same time endogenous protein were biotinylated by adding 500 μM biotin, 1 mM ATP and 5 mM MgCl_2_ for 15 min. Labeling was stopped by placing cells on ice and washing six times with ice-cold PBS. Then the cells were detached in ~ 1.5 mL RIPA lysis buffer (50 mM Tris pH 8, 150mM NaCl, 0.1% SDS, 0.5% sodium deoxycholate, 1% Triton X-100, protease inhibitor cocktail, and 1 mM phenylmethylsulfonyl fluoride) from the dish using a cell scraper, and incubated for 30 min on ice. Lysates were clarified by centrifugation at 12,000× g for 10 min at 4°C. In order to enrich biotinylated proteins from the lysates, 200 μL streptavidin-coated magnetic beads (Pierce) were washed twice with RIPA buffer, and incubated with clarified lysates of each sample with rotation for 1 h at room temperature, then moved and incubated at 4°C overnight. The beads were subsequently washed twice with1 mL of RIPA lysis buffer, once with 1 mL of 1 M KCl, once with 1 mL of 0.1 M Na_2_CO_3_, once with 1 mL of 2 M urea in 10 mM Tris-HCl (pH 8.0), and twice with 1 mL RIPA lysis buffer, and then were shipped to Shanghai Omicsolution Co., Ltd. with dry ice for further LC-MS/MS analysis. The data of mass spectrometry were analyzed as previously described, and the result was shown in **Supplementary Table S1**. A volcano plot was adopted to illustrate the differentially biotinylated proteins, which was drawn with the ggplot2 package in R.

### 2.5 Western blotting and co-immunoprecipitation

Whole-cell lysates for both WB and Co-IP were prepared using the lysis buffer containing 50 mM Tris-base (pH 7.5), 1 mM EGTA, 1 mM EDTA, 1% Triton X-100, 150 mM NaCl, 100 µM phenylmethylsulfonyl fluoride (PMSF) and protease inhibitor cocktail (Roche) at 4°C for 30 min. Then the cell lysates were centrifuged at 14,000×g at 4°C for 10 min. For WB, the supernatants were mixed well with sample buffer and followed by denaturation at 95°C for 10 min. For Co-IP, the supernatants were collected and mixed with Protein G-agarose (Millipore) and anti-HA antibodies (2μg, Sigma Aldrich) for 16 h at 4°C. After washed six times with ice-cold lysis buffer, Protein G agarose-bound immune complexes were then eluted by sample buffer and subjected to WB analysis. And the denatured samples were resolved by SDS-PAGE and transferred to nitrocellulose membranes, then the membranes were blocked with TBST (pH 7.4, containing 0.1% Tween-20) containing 5% skimmed milk for 1 h at room temperature, followed by incubation with primary antibodies to ACE2 (1:1000, Proteintech), biotin (1:1000, Cell Signalling Technology), HA (1:1000, Sigma Aldrich), SARS-CoV-2 S (1:1000, ABclonal) and actin (1:1000, Proteintech) at 4°C overnight. After, the membranes were washed and incubated with the HRP-conjugated secondary antibodies for 1 h at room temperature and imaged using the FluorChem HD2 system (Alpha Innotech). Images were analyzed using AlphaEaseFC software (Alpha Innotech).

### 2.6 Pseudovirus binding assay

For the pseudovirus binding assay, A549 cells were washed twice with ice-cold PBS and incubated with SARS-CoV-2 pseudovirus at an MOI of 20 in cold DMEM medium supplemented with 2% FBS on ice for 1 h. Afterwards the supernatant was removed and the cells were washed six times with ice-cold PBS. Then the cells were collected and the copies of SARS-CoV-2 N gene were measured by quantitative PCR to evaluate absorption of pseudovirus.

### 2.7 Inhibitor assay

Dubermatinib (TP0903) is a potent and selective AXL receptor tyrosine kinase inhibitor, purchased from TOPSCIENCE. 1×10^5^ A549 cells were preincubated with serially diluted Dubermatinib for 2 h at 37°C. Then 2×10^5^ IFUs of SARS-CoV-2 pseudovirus (MOI of 2) were added and incubated at 37°C for 1 h, and the cells were washed with PBS and cultured in fresh medium at 37°C for another 48 h. Cells were collected and the copies of SARS-CoV-2 N gene were measured by quantitative PCR.

### 2.8 Quantitative PCR

Total cellular RNA was isolated with TRIzol (Invitrogen) reagent according to the manufacturer’s protocols. The quantification of SARS-CoV-2 N gene was analyzed by one-step real-time qPCR with the HiScript II One Step qRT-PCR SYBR Green Kit (Vazyme) using specific primers and the Applied Biosystems ViiA7 real-time PCR system. The data were normalized to the levels of β-actin in each individual sample.

### 2.9 Statistical analysis

The data were analyzed and presented as the mean ± standard error (SEM) using GraphPad 8.0. Statistical significance between two groups was determined using two-tailed unpaired Student’s t-test. Differences were considered to be significant for p value < 0.05. For differential biotinylated protein analysis, proteins with the p value < 0.05 and |log2(fold change)| > 1 were considered to be the differential biotinylated proteins.

## 3 Results

### 3.1 Construction and validation of lentiviral plasmid carrying ACE2 fusing TurboID

**Figure 1:**
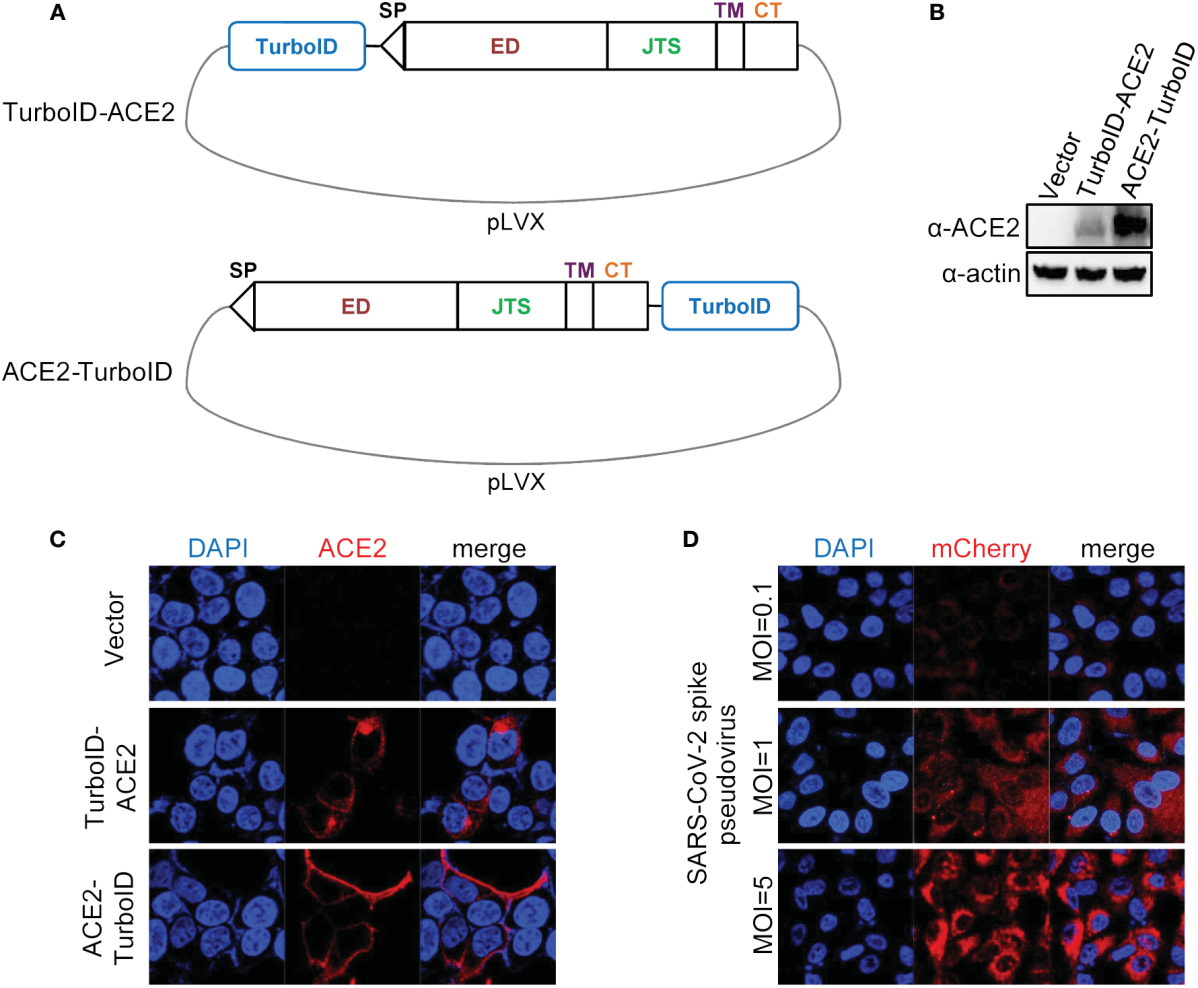
Construction and validation of lentiviral plasmid carrying ACE2 fusing TurboID. **(A)** Diagram of construction of ACE2-TurboID and TurboID-ACE2 overexpression lentiviral plasmid. Signal peptide (sp), ectodomain (ED), juxtamembrane stalk (JTS), transmembrane domain (TM), cytosolic tail (CT), TurboID, promiscuous biotin ligase with 15 mutations relative to wild-type BirA. **(B, C)** A549 cells were transduced with lentiviruses carrying the TurboID-ACE2 or ACE2-TruboID, respectively. At 72 h post infection, the protein levels of TurboID-ACE2 and ACE2-TruboID were determined by western blot analysis **(B)**. For immunofluorescence analysis, the cells were plated on the glass dishes for another 24 h, and then fixed with paraformaldehyde and stained using the anti-ACE2 antibody, and finally analyzed by confocal microscopy **(C)**. **(D)** A549 cells stably overexpressing ACE2-TurboID were infected with SARS-CoV-2 spike pseudotyped lentivirus bearing mCherry-SARS-CoV-2 N gene. At 48 h post infection, the level of mCherry-N was observed with the confocal microscopy.

We first constructed a plasmid carrying ACE2 coupled with the TurboID gene, and we adopted two schemes to construct this plasmid, one was to fuse the TurboID gene to the 5’ end of the ACE2 gene, and the other fuse the TurboID gene to the 3’ end of the ACE2 gene (**Figure 1A**). After co-transfecting these two transfer plasmids with lentiviral packaging plasmids into HEK293T cells and obtaining overexpression lentiviruses, the effectiveness of these two kinds of lentiviruses was verified by infecting A549 cells. Both of the results of immunoblotting (**Figure 1B**) and immunofluorescence (**Figure 1C**) showed that fusing the TurboID gene to the 3’ end of the ACE2 gene was more conducive to the expression of the ACE2 gene, and this construction strategy also could allow ACE2 to be correctly located on the cell membrane. Furthermore, ACE2-TurboID also mediated SARS-CoV-2 pseudovirus to efficiently infect A549 cells (**Figure 1D**). The subsequent PL was performed using A549 cells stably overexpressing ACE2-TurboID.

### 3.2 Identification of SARS-CoV-2 candidate co-receptors by PL screen

**Figure 2:**
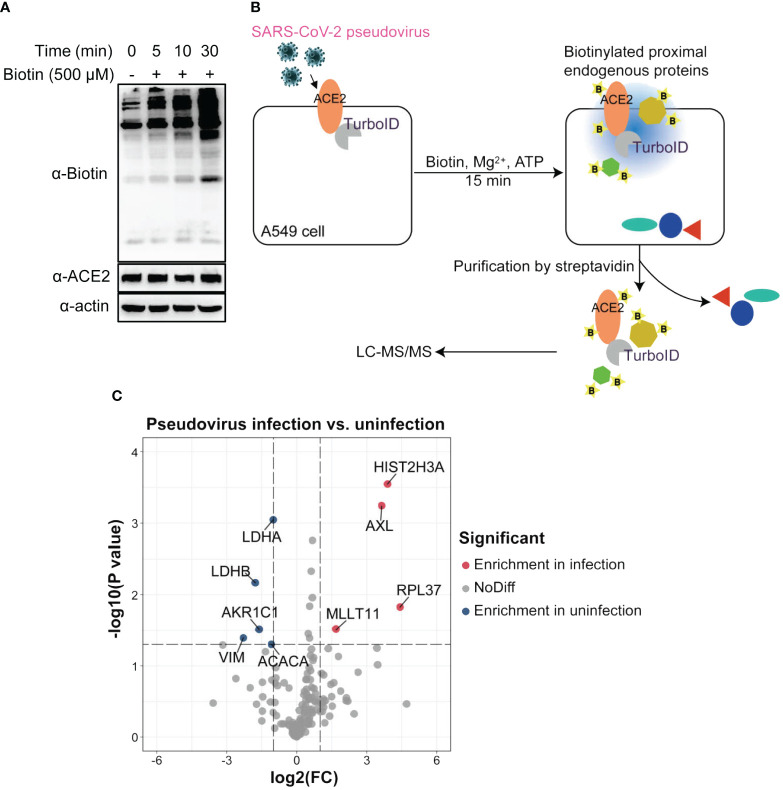
Identification of SARS-CoV-2 candidate co-receptors by proximity labeling screening. **(A)** Detection of the enzymatic activity of ACE2-TurboID and determination of the optimal reaction time for proximity labeling. A549 cells stably overexpressing ACE2-TurboID were incubated with 500 μM biotin and reaction buffer and collected at indicated times, followed by western blot analysis. **(B)** Workflow of proximity labeling. A549 cells stably overexpressing ACE2-TurboID were infected with SARS-CoV-2 spike pseudovirus at an MOI of 2 or left uninfection for 15 min accompanied by proximity labeling. Then the cells were harvested and purified by streptavidin, followed by LC-MS/MS analysis. **(C)** Volcano plot demonstrating the differentially biotinylated proteins in infection and uninfection groups. The x-axis stands for the log 2 fold change (infection/uninfection) while the y-axis stands for the value of -log 10 of p value. The differentially biotinylated proteins are indicated in red and blue (p < 0.05 & |log2(FC)| > 1).

In order to perform PL, we first verified the biotinylation activity of ACE2-TurboID. After addition of biotin, ATP and MgCl_2_, A549 cells overexpressing ACE2-TurboID could efficiently biotinylate endogenous proteins in a short time (**Figure 2A**). Subsequently, the cells were inoculated with SARS-CoV-2 pseudovirus for 15 min accompanied by PL in the presence of the biotin, ATP and MgCl_2_. The biotinylated proteins were enriched using streptavidin-coated beads, and identified by mass spectrometry (**Figure 2B**). The volcano plot showed that 4 proteins, namely HIST2H3A, AXL, RPL37 and MLLT11, were significantly enriched in the SARS-CoV-2 pseudovirus infection group (**Figure 2C**; **Supplementary Table S1**). However among them, AXL was the only membrane protein, and in general, virus co-receptors are membrane proteins (Chappell and Dermody, 2015; Maginnis, 2018; Zhang et al., 2019). Thus, we suggested that AXL might be a candidate co-receptor for SARS-CoV-2 to enter A549 cells.

### 3.3 AXL facilitates SARS-CoV-2 entry into host cells

**Figure 3:**
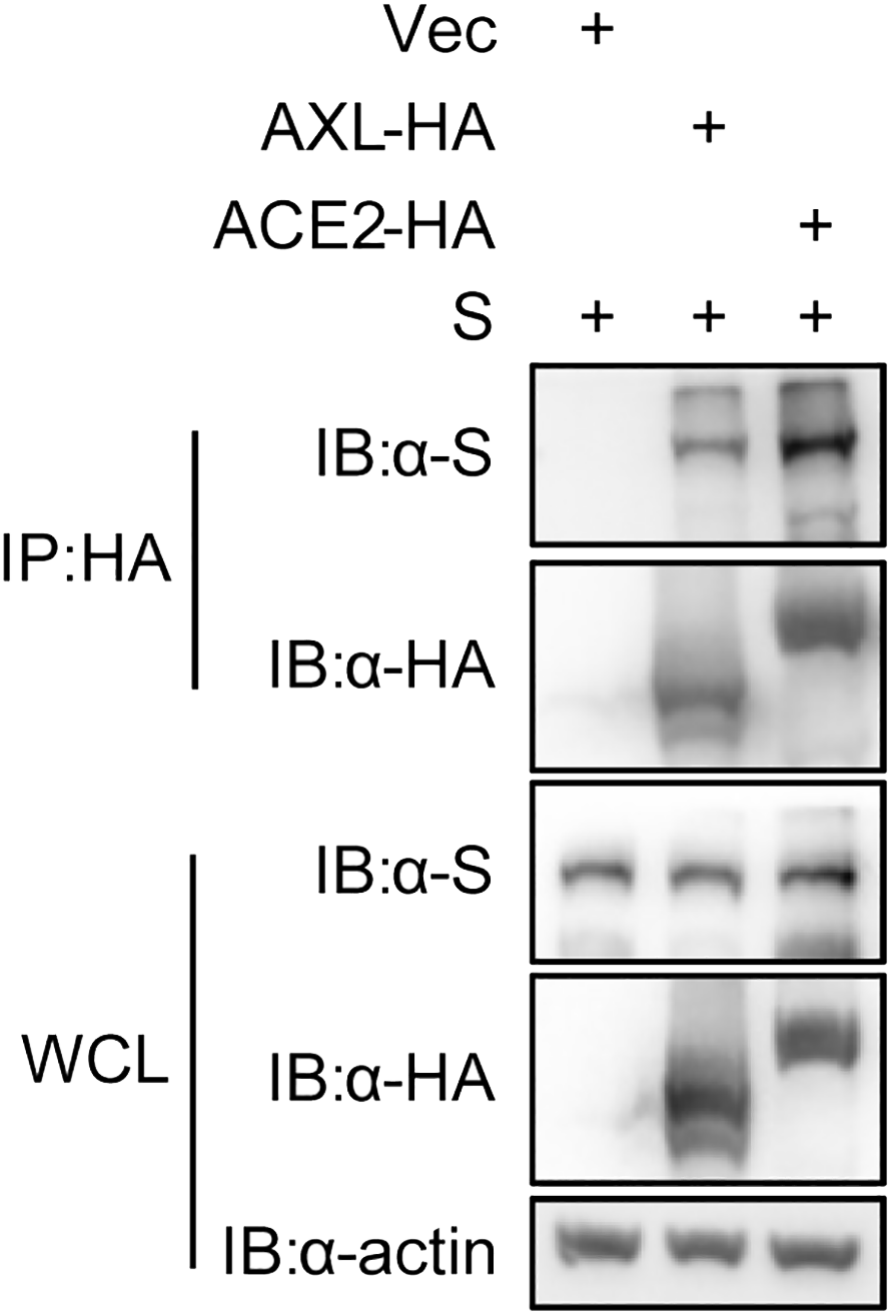
SARS-CoV-2 S interacts with AXL. HEK293T cells were co-transfected with ACE2-HA or AXL-HA and SARS-CoV-2 S, respectively. At 48 h post transfection, the cells were lysed and HA-tagged proteins were immunoprecipitated with anti-HA antibody. Inputs and precipitated proteins were determined by western blotting with the indicated antibody.

**Figure 4:**
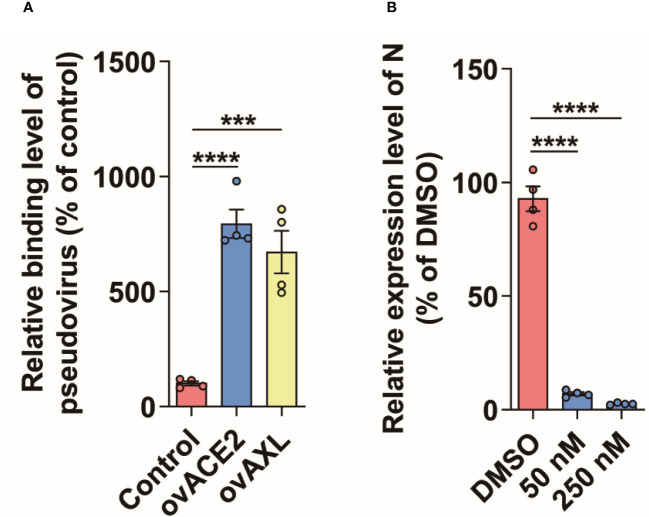
AXL facilitates SARS-CoV-2 entry into host cells. **(A)** SARS-CoV-2 pseudovirus carrying mCherry-SARS-CoV-2 N was incubated with indicated cells at 4°C for 1 h (n=4), then the cells were collected and the copies of N were measured by qPCR using specific primers of N gene. **(B)** A549 cells stably overexpressing AXL were preincubated with serially diluted Dubermatinib for 2 h and then were infected with SARS-CoV-2 pseudovirus carrying mCherry-N for another 48 h at 37°C (n=4), followed by the measurement of the relative expression level of N in the cells. Quantitative data in this figure are shown as the mean ± SEM. ***p< 0.001, ****p< 0.0001.

The proteins labeled by TurboID may be only close to the target protein, and not interact with the target protein (Yang et al., 2021). To further rule out this possibility, we used the immunoprecipitation to verify the results observed with PL. Like ACE2, AXL was indeed able to immunoprecipitate with the S protein of SARS-CoV-2 (**Figure 3**). Moreover, both the virus binding assay (**Figure 4A**) and the AXL inhibitor assay (**Figure 4B**) confirmed the possibility of AXL as a receptor for SARS-CoV-2 entry.

## 4 Discussion

In present study, we performed a PPI screening for host factors required for SARS-CoV-2 entry using the TurboID-catalyzed PL system. The biotinylated proteins were identified by LC-MS/MS, and AXL was significantly enriched in cells infected with SARS-CoV-2 pseudovirus. Further functional analysis confirmed that AXL was a mediator of SARS-CoV-2 entry into host cells. This finding was also reported by Wang and colleagues (Wang et al., 2021). To the best of our knowledge, this is the first study to use PL to identify virus receptors/co-receptors, and in turn our data also illustrate the effectiveness of PL for the identification of virus receptors.

The ability of viruses to cause productive viral infections and to spread within organisms is largely dependent on the recognition of receptors and other co-receptors expressed on the surface of susceptible host cells (Husain et al., 2022). Cellular receptors are important targets for antiviral strategies, and the discovery and identification of functional virus receptors is essential for a better understanding of the virus entry mechanism and the prevention and control of viral diseases. Currently, the most commonly used method for virus receptor identification is affinity purification coupled to mass spectrometry (AP-MS) (Zapatero-Belinchón et al., 2021), which has been used to identify the key receptors of a variety of important viruses, such as the receptor sodium taurocholate co-transporting polypeptide (NTCP) of hepatitis B and D viruses (Yan et al., 2012), the receptor neural cell adhesion molecule NCAM1 of Zika virus (Srivastava et al., 2020), and the SARS-CoV-2 receptor AXL identified by Wang et al (Wang et al., 2021). AP-MS typically requires stable interactions, but some non-specifically bound proteins will inevitably be purified during the purification process. For example, in the process of identifying the SARS-CoV-2 receptor AXL, Wang et al. initially identified 2153 proteins after two purifications, and then reduced the number of candidate proteins to 3 by combining other methods. While we initially identified only 189 high-confidence proteins using TurboID-catalyzed PL, which greatly simplified the data screening process compared to AP-MS. This may be related to the high spatial specificity of PL or to the fact that PL is performed in live cells, whereas AP-MS is performed in cell lysates, which are usually not spatially specific. Gingras AC et al. argue that PL is generally limited to in-cis interactions that take place in a cell membrane (Gingras et al., 2019; Husain et al., 2022), but a PL technique called PUT-IT, described by Liu Q et al., has also shown feasibility in identifying in-trans interactions (Liu et al., 2018). Therefore, the pseudovirus with an extracellular domain of viral membrane protein coupled to a PL enzyme could be used for the identification of novel virus receptors. In addition, in order to improve the specificity of the affinity purification process, AP-MS usually requires the use of tandem tags and multistep protein purification, which is more complex than the TurboID-catalyzed PL.

In addition to the above advantages, there may be some technical limitations of PL technology. To improve the specificity of PL, we chose PL enzymes with short labeling times, and virus entering into cells is a multi-step, multi-factorial process, which may result in a subset of late co-receptors not being identified. On the contrary, if a PL enzyme (such as BioID) with a long labeling time was used, it might lead to a decrease in labeling specificity and increase the subsequent screening process of the authentic receptor, so it is necessary to make a reasonable technical selection according to the actual needs. Further, more than 5 SARS-CoV-2 receptors have been identified so far, and no other known receptors, such as ASGR1, KREMEN1, CD147, NRP1, etc (Gu et al., 2022), have been identified by PL technology, and the same phenomenon exists in other research publications of SARS-CoV-2 receptors, that is, there is little overlap between SARS-CoV-2 receptors identified by different research teams using different methods. The reasons for this phenomenon may be multifactorial: SARS-CoV-2 exhibits a wide range of tissue tropism, and different entry strategies may be used for different tissues, such as ASGR1, a receptor for SARS-CoV-2 infection in the liver (Yang et al., 2024), which is highly expressed in the liver and almost not expressed in A549 cells, or it may be due to objective differences in the detection of ligand-receptor interactions between cell-based screening methods and co-immunoprecipitation-based methods. Specifically for our study, the initial goal was to identify ACE2-dependent SARS-CoV-2 co-receptors, and short-term labeling only identified strongly interacting proteins, and AXL protein levels were high in A549 cells, which may have resulted in other receptors not being identified. There is a need for further validation of the application of PL technology for virus receptors or co-receptors identification.

Meanwhile viruses often use multiple receptors or co-receptors in order to establish an efficient infection process and to adapt to changes in different cell types. These receptors and co-receptors can provide more opportunities and pathways for the virus to interact with the cell surface, thereby increasing the success rate of infection. During HIV entry, the virus needs to bind not only to CD4, but also to co-receptors such as CCR5 or CXCR4 to successfully enter the host cell (Mercer et al., 2020). This multiple receptor and co-receptor dependent infection strategy is often multi-step, complex and transient, but there is currently no technology that can accurately elucidate this complex process, and time-sensitive PL offers a novel approach to this problem. In conclusion, our studies demonstrate the feasibility of PL for virus receptors or co-receptors identification.

## Data availability statement

The datasets presented in this study can be found in online repositories. The names of the repository/repositories and accession number(s) can be found in the article/**Supplementary Material**.

## Author contributions

BW: Conceptualization, Data curation, Formal analysis, Investigation, Methodology, Project administration, Resources, Supervision, Writing – original draft, Writing – review & editing. FY: Formal analysis, Writing – review & editing. WW: Formal analysis, Writing – review & editing. FZ: Visualization, Writing – review & editing. XS: Conceptualization, Methodology, Project administration, Resources, Supervision, Writing – original draft, Writing – review & editing.

## Supplementary material

Supplementary Table 1 The result of LC-MS/MS.
